# Impact of Healthcare Non-Take-Up on Adherence to Long-Term Positive Airway Pressure Therapy

**DOI:** 10.3389/fpubh.2021.713313

**Published:** 2021-08-17

**Authors:** Najeh Daabek, Renaud Tamisier, Alison Foote, Hélèna Revil, Marie Joyeux-Jaure, Jean-Louis Pépin, Sébastien Bailly, Jean-Christian Borel

**Affiliations:** ^1^HP2 Laboratory, INSERM U1042, University Grenoble Alpes, Grenoble, France; ^2^AGIR à dom. Homecare Charity, Meylan, France; ^3^EFCR Laboratory, Grenoble Alpes University Hospital, Grenoble, France; ^4^Research Division, Grenoble Alpes University Hospital, Grenoble, France; ^5^Social Sciences Research – PACTE Laboratory, CNRS UMR 5194, University Grenoble Alpes, Grenoble, France

**Keywords:** CPAP, non-invasive ventilation, PAP therapy, healthcare non take up, adherence—compliance—persistence

## Abstract

**Background:** The effectiveness of positive airway pressure therapies (PAP) is contingent on treatment adherence. We hypothesized that forgoing healthcare may be a determinant of adherence to PAP therapy.

**Research Question:** The objectives were: (i) to assess the impact of forgoing healthcare on adherence to PAP in patients with Chronic Respiratory Failure (CRF) and patients with Obstructive Sleep Apnea Syndrome (OSAS); (ii) to compare forgoing healthcare patterns in these two chronic conditions.

**Study design and methods:** Prospective cohort of patients with OSAS or CRF, treated with PAP therapies at home for at least 12 months. At inclusion, patients were asked to fill-in questionnaires investigating (i) healthcare forgone, (ii) deprivation (EPICES score), (iii) socio-professional and familial status. Characteristics at inclusion were extracted from medical records. PAP adherence was collected from the device's built-in time counters. Multivariable logistic regression models were used to assess the associations between healthcare forgone and the risk of being non-adherent to CPAP treatment.

**Results:** Among 298 patients included (294 analyzed); 33.7% reported forgoing healthcare. Deprivation (EPICES score > 30) was independently associated with the risk of non-adherence (OR = 3.57, 95%CI [1.12; 11.37]). Forgoing healthcare had an additional effect on the risk of non-adherence among deprived patients (OR = 7.74, 95%CI [2.59; 23.12]). OSAS patients mainly forwent healthcare for financial reasons (49% vs. 12.5% in CRF group), whereas CRF patients forwent healthcare due to lack of mobility (25%, vs. 5.9 % in OSAS group).

**Interpretation:** Forgoing healthcare contributes to the risk of PAP non-adherence particularly among deprived patients. Measures tailored to tackle forgoing healthcare may improve the overall quality of care in PAP therapies.

**Clinical Trial Registration:** The study protocol was registered in ClinicalTrials.gov, identifier: NCT03591250.

## Introduction

Sleep breathing disorders, particularly obstructive sleep apnea syndrome (OSAS), nocturnal alveolar hypoventilation and at worst chronic respiratory failure (CRF) are associated with incapacitating symptoms affecting quality of life, and poor long term outcomes including cardio-vascular events and early mortality (1–3). Since the early 80s', non-invasive positive airway pressure therapies [Continuous Positive Airway Pressure (CPAP) and Non-Invasive Ventilation (NIV)] have been the first-line treatments for OSAS and CRF (4–7).

The effectiveness of positive airway pressure (PAP) therapies is however contingent on treatment adherence, and a significant proportion of non-adherence and high therapy termination rates are observed (8, 9). Despite continuous technological innovations, adherence to PAP therapies has plateaued over the last 20 years (10), suggesting that adherence is dependent on patients' personal characteristics such as their marital or social status (11–13), their perception of treatment efficacy (14), any benefits experienced (15), and their priorities regarding personal lifestyle (16–18).

Poor adherence to PAP therapies might reflect societal vulnerability, deprivation and non-prioritization of personal health. A comprehensive and holistic way of investigating and understanding health-related behaviors is to study reasons individuals forgo healthcare and to estimate the prevalence of this attitude. The concept of forgoing healthcare corresponds to societal, health-system contexts or personal conducts and/or beliefs leading individuals to forgo or postpone self-identified healthcare needs to which they have rights. This concept allows us to understand the relationship that people have with the healthcare system and to apprehend the influence of individual and collective factors on health related behavior. A large part of research on the forgoing healthcare phenomenon has focused on underprivileged populations who forgo healthcare primarily for financial reasons (19, 20).

However, multiple reasons for forgoing healthcare are also reported by individuals without financial constraints. These include lack of time owing to the burden of professional or personal life, lassitude or negligence, and inadequate transport with long distances between their residence and care facilities. In addition, some studies on the concept of forgoing healthcare show that not all people are exposed in the same way to this phenomenon. Depending on their sex, family and/or professional situation, or their level of multidimensional deprivation, the pattern of forgoing healthcare varies (21). Furthermore, qualitative social science studies indicate that individuals can forgo care related to a particular chronic condition but seek treatment for other conditions and vice versa (22).

Therefore, assessing influence of socioeconomics factors like deprivation and healthcare non-take up on specific populations like OSAS and CRF patients is an essential step to personalization and optimization of the healthcare delivery. In addition, unlike oral treatments, PAP therapies required for OSA and CRF patients have the advantage of a long-term objective assessment of treatment adherence (thanks to telemonitoring). These respiratory pathologies represent therefore an ideal disease model for designing and testing multifactorial interventions to promote treatment adherence. Moreover, OSA and CRF subgroups have well-known differences in clinical presentations and socio-economic status that could generate different profiles for health care renunciation.

In this study, we hypothesized that forgoing healthcare may be a significant determinant of PAP-therapies adherence. As, clinical presentation and socioeconomic status is dissimilar between OSAS and CRF populations, we decided to evaluate and compare the prevalence of forgoing healthcare (related or not to their respiratory disease) in two populations, OSAS and CRF patients, both on long-term home PAP treatment. We compared the ways in which individuals forwent healthcare and the reasons.

## Materials and Methods

### Study Design

The present study was a prospective monocentric cohort study (Department of Pulmonology, Grenoble Alpes University Hospital). Ethical approval was obtained from the French Ethics Committee “Ile de France II” and the study protocol was registered in ClinicalTrials.gov (NCT03591250). The study was conducted between June 2018 and November 2019. Each participant provided written informed consent before inclusion in the study.

### Study Participants

During a routine medical follow-up consultation, patients meeting the following inclusion criteria were asked to participate (Supplementary Figure 1):

- Age above 18 years
- Affiliated to the French social security system or a beneficiary of this system
- A diagnosis of OSAS or CRF
- Treated with CPAP or NIV for at least 12 months
- Routinely followed by the same homecare provider (AGIR à Dom, Meylan, France)
- Able to fill in the study questionnaires.

### Study Objectives

Our primary objective was the impact of forgoing healthcare on adherence to PAP therapy. The secondary objective was a comparison of forgoing healthcare patterns between patients with CRF and patients with OSAS.

### Data Collection and Procedures

#### Assessment of Healthcare Forgone

Participants were asked to fill-in the “healthcare non-take up” questionnaire during their routine medical follow-up consultation in the Department of Pulmonology, Grenoble Alpes University Hospital. This questionnaire was originally developed by Dr. Revil's group at the PACTES laboratory (Grenoble-Alpes University, France); and previously used by us in a study of 164,092 public sector health insurance beneficiaries in France (23). Briefly, the questionnaire is structured into three sections and refers to healthcare forgone in the 12 months preceding the study inclusion consultation (Supplementary Figure 2):

i. *Healthcare forgone*: After the key question “Have you forgone or put-off healthcare on one or more occasions in the last 12 months (yes/no),” those answering “yes” were asked about the type(s) of healthcare forgone and their reasons, how long they had been forgoing or putting-off healthcare and their perception of their current state of health.
ii. *Healthcare insurance*: This section focused on whether participants had complementary, top-up health insurance [through a private company or the state-subsidized “Complémentaire Santé Solidaire” (CSS)]; and if not, the reasons why. They were also asked whether they benefited from 100% cover by the state system due to a long-term chronic condition (e.g., Type I diabetes).

*Briefly, France has a two-tier system of health insurance: a compulsory primary health insurance scheme and complementary/top-up health insurance schemes. In the compulsory scheme, contributions are proportional to income and reimbursement of care is a fixed percentage of the total cost of care. The rate of reimbursement is set by the state and depends on the type of care. Complementary schemes are essentially private insurance policies which reimburse almost all the remaining healthcare costs not covered by the compulsory scheme. However, for people on low incomes, a means-tested top-up scheme is provided by the state; this “Complémentaire Santé Solidaire” (CSS) is free of charge. Finally, the compulsory French state scheme covers 100% of health expenses related to 29 severe chronic diseases including diabetes, chronic respiratory failure, cancer, cystic fibrosis etc. The list of eligible conditions is set by the public health code*.

iii. *Standard of living and deprivation:* Socio-professional and familial status were collected. Material and social deprivation were investigated using the 11 item EPICES questionnaire (24, 25). An individual score was calculated for each participant, by adding each question coefficient to the intercept whenever the answer is “yes.” According to EPICES a score of ≥ 30 indicates deprivation.

#### Clinical Data and Other Socio-Demographics

Characteristics at inclusion, including age, sex, anthropometrics, main etiologies of respiratory disorders, and hospitalization in the year before inclusion were extracted from the participants' medical records. Data related to NIV or CPAP: date of treatment initiation, PAP adherence in the year following inclusion in the study (objectively measured from the device's built-in time counters and reported every 6 months) and type of mask, were collected from the homecare provider's database.

#### Sample Size

Based on the hypothesis of a 25% prevalence of forgoing healthcare in the population (23), and allowing for 10% dropout, the enrollment of 300 participants (150 patients treated with CPAP; 150 treated with NIV) would allow 80% power to detect a difference of 1.5 ± 3 (SD) hours/night in CPAP/NIV adherence between patients who forwent healthcare and those who did not.

### Statistical Analysis

Descriptive statistics are presented as medians [IQR] for quantitative variables and frequencies (%) for qualitative variables. Chi square tests and non-parametric Mann-Whitney tests were used to compare qualitative and quantitative variables, respectively, between groups (OSAS vs. CRF).

A simple imputation method was used in cases with little missing data (<2%) (26). Otherwise, multiple imputation with fully conditional specification was performed (27).

#### Primary Outcome Analysis

Average PAP therapy use was defined as the mean of the measures collected from the devices in the year following inclusion. Normality of mean PAP use was assessed both graphically and using the Shapiro-Wilk test, and was not accepted. Thus, data were dichotomized using a threshold of 4 h/night and the adherence to PAP therapy was defined as: adherent for an average of ≥4 h/night, and otherwise non-adherent.

To identify whether forgoing healthcare impacted adherence to PAP therapies, univariable logistic modeling was performed. Covariates were chosen *a priori* based on factors that might impact PAP-therapy adherence, and included sex (28), age, BMI, family and socio-professional status (11, 29) complementary healthcare insurance, reimbursement rates, healthcare forgone, degree of deprivation (EPICES score) (30), PAP-therapy duration (years), number of hospitalizations, and etiology. Variables with a *p* < 0.25 were then introduced into a multivariable model. Given that we were not looking for a predictive model (therefore no evaluation based on performance) but an explanatory model, and in order to take into account the potential confounding factors, a stepwise descending selection was used for the final model selection.

Given the collinearity between forgoing healthcare and deprivation, a four-modality categorical variable was used in the model: (1) healthcare forgone and no deprivation, (2) no healthcare forgone and deprivation, (3) healthcare forgone and deprivation, (4) no deprivation and no healthcare forgone.

#### Secondary Outcome Analysis

A comparison of the pattern of forgoing healthcare between patients with OSAS and those with CRF was conducted using a Chi-square test for qualitative variables and a non-parametric Wilcoxon test for the quantitative variables.

## Results

The study flow chart is shown in Supplementary Figure 3. Of 298 patients included in the study and who responded to the healthcare non-take-up questionnaire, four patients had no objective measure of their PAP adherence and were excluded from the analysis. None of the patients refused to fill-in the questionnaire.

Table 1 shows the main characteristics of the study population. Participants were predominantly male (64.3%) and obese (30.8 [25.4; 35.4] kg/m^2^). Large proportions of the study cohort were living as a couple (67.1%) and/or retired (61.8%). All participants with CRF were prescribed treatment with NIV and 93.7% of OSAS patients were prescribed CPAP at night. Median adherence to PAP therapy was high (7.3 h [5.4; 8.8]) with only 12.8% non-adherent patients, i.e., under the 4 h/night threshold. There was no difference in treatment adherence between CRF and OSAS patients.

**Table 1:** General and clinical characteristics of patients (*N* = 294).

**Variable**	**Items**	**Whole population (*N* = 294)**	**OSAS (*N* = 158, 53.74%)**	**CRF (*N* = 136, 46.26 %)**	*p*-value*	**Missing**
BMI (Kg/m^2^)		30.8 [25.4; 35.4]	31.1 [26.6; 34.8]	29.4 [23.3; 37.3]	0.40	7
Sex	M	189 (64.3)	126 (79.7)	63 (46.3)	< 0.01	0
Age	≤60 years	83 (28.2)	43 (27.2)	40 (29.4)	0.41	0
	]60;70]	109 (37.1)	64 (40.5)	45 (33.1)		
	>70 years	102 (34.7)	51 (32.3)	51 (37.5)		
Family situation	Couples	160 (55.4)	104 (67.1)	56 (41.8)	< 0.01	5
	Alone	129 (44.6)	51 (32.9)	78 (58.2)		
Socio-professional status	Working	66 (22.8)	48 (30.8)	18 (13.4)	< 0.01	4
	Retired or Unemployed	224 (77.2)	108 (69.2)	116 (86.6)		
PAP Therapy	CPAP	148 (50.3)	148 (93.7)	0 (0)	< 0.01	0
	NIV	146 (49.7)	10 (6.3)	136 (100)		
Indication for PAP therapy	OSAS	157 (53.4)	157 (99.4)	0 (0)	< 0.01	0
	COPD	26 (8.8)	0 (0)	26 (19.1)		
	Neuromuscular pathology	23 (7.8)	0 (0)	23 (16.9)		
	OHS	32 (10.9)	1 (0.6)	31 (22.8)		
	Chest well disorder and others	56 (19)	0 (0)	56 (41.2)		
Delay since PAP therapy initiation (years)		7.2 [2.2; 12.2]	7.9 [2.9; 13]	5.2 [1.7; 10.9]	0.02	0
Delay since primary diagnosis (years)		8.5 [4.6; 12.9]	8.4 [3.4; 12.5]	10.8 [6.4; 14.7]	0.08	137
Number of hospitalizations in the year preceding inclusion		0 [0; 2]	0 [0; 0]	2 [0; 4]	< 0.01	0
% of patients with PAP adherence >4 h/night	Yes	232 (87.2)	134 (87.6)	98 (86.7)	0.84	28
Average PAP-therapy adherence (h/night)		7.3 [5.4; 8.8]	6.8 [5.3; 8]	8.2 [5.8; 10.4]	< 0.01	28

*Values in Numbers (%) or median [IQR]*.

* *Comparaison of CRF and OSAS groups (p-value were calculated using Chi square tests and non-parametric Mann-Whitney tests)*.

*BMI, Body mass index; COPD, Chronic obstructive pulmonary disease; CPAP, Continuous positive airway pressure; CRF, Chronic respiratory failure; NIV, Non-invasive ventilation; OHS, Obesity hypoventilation syndrome; OSAS, Sleep apnea syndrome; PAP, Positive airway pressure*.

Over a third of the population (33.7%) declared forgoing at least one item of healthcare in the 12 months preceding inclusion and 53.4 % were considered to be deprived (EPICES score > 30) (Table 2). Patients with CRF were more often covered by health insurance at a rate of 100% than patients with sleep apnea (88.8% vs. 39.7%, respectively, *p* < 0.01) and were thus more often exempted from expenses related to their chronic illness (88.8 vs. 39.1, respectively; *p* < 0.01).

**Table 2:** Access to care, healthcare coverage, and deprivation (***N*** = 294).

**Variable**	**Items**	**All population (*N* = 294)**	**OSAS (*N* = 158, 53.74%)**	**CRF (*N* = 136, 46.26 %)**	***p*-value**	**Miss**
**Access to care**						
Health care forgo	Yes	99 (33.7)	51 (32.3)	48 (35.3)	0.59	0
**Healthcare coverage**						
Coverage ratio	Partial (60%)	109 (37.6)	94 (60.3)	15 (11.2)	< 0.01	4
	100%	181 (62.4)	62 (39.7)	119 (88.8)		
100% coverage due to total disability or long-term illness	Yes	180 (62.1)	61 (39.1)	119 (88.8)	< 0.01	4
Complementary (top-up) health insurance	None or state-subsidized “Complémentaire Santé Solidaire (CSC)”	46 (15.8)	18 (11.4)	28 (20.9)	0.03	2
	Private	246 (84.2)	140 (88.6)	106 (79.1)	.	.
**Reasons for not having top-up insurance**						
-“I don't see the point”	Yes	2 (14.3)	2 (50)	0 (0)	0.02	0
-“I am 100% covered by Health Insurance and I don't think I need any additional”	Yes	7 (50)	1 (25)	6 (60)	0.24	0
**Deprivation**						
EPICES score		31.4 [15.4; 47.3]	23.1 [10.1; 42.6]	37.3 [26.6; 50]	< 0.01	0
Patients with EPICES score >30	N (%)	157 (53.4)	69 (43.7)	88 (64.7)	< 0.01	0
**Deprivation and healthcare non-take up**						
No deprivation and no healthcare non-take up		111 (37.8)	40 (29.41)	71 (44.94)	< 0.01	0
Deprivation and no healthcare non-take up		84 (28.6)	48 (35.29)	36 (22.78)		
No deprivation and healthcare non-take up		26 (8.8)	8 (5.88)	18 (11.39)		
Deprivation and healthcare non-take up		73 (24.8)	40 (29.41)	33 (20.89)		

*Values in Numbers (%) or median [IQR]. CRF, Chronic respiratory failure; OSAS, Sleep apnea syndrome*.

Concerning the Impact of forgoing healthcare on adherence to PAP therapies: univariable analysis between adherence to PAP therapies and the different variables of interest are presented in Supplementary Table 1. In multivariable analysis, deprivation (EPICES score >30) was independently associated with the risk of being non-adherent (OR = 3.57, 95%CI [1.12; 11.37], *p* = 0.031). We were not able to demonstrate an independent association between healthcare non take up and PAP therapy adherence, however forgoing healthcare had an additional effect on the risk of non-adherence among patients experiencing deprivation (OR = 7.74, 95%CI: [2.59; 23.12], *p* < 0.001) (Table 3).

**Table 3:** Multivariable association between predictors and the probability of being non-compliant (***N*** = 266).

**Variable**	**Items**	**OR**	**95% CI**	***p*** **-value**
Deprivation and healthcare non-take up	Deprivation and no healthcare non-take up	3.57	[1.12; 11.37]	0.0311
	Deprivation and healthcare non-take up	2.01	[0.35; 11.68]	0.4332
	Deprivation and healthcare non-take up	7.74	[2.59; 23.12]	0.0002
	Deprivation and no healthcare non-take up (reference)			
Time since initiation of PAP-Therapy		0.88	[0.81; 0.95]	0.0021
Hospitalizations in the previous year	≥1 hospitalization	0.40	[0.17; 0.96]	0.0395
	No hospitalization			

*An univariable analysis was conducted to select the variablesto be included in the multivariable analysis, which were: The reimbursement rate, PAP-therapy duration (years), number of hospitalizations in the previous year and the interaction between healthcare non-take up and deprivation*.

Longer time since PAP-therapy initiation was significantly associated with a lower probability of being non-adherent (OR = 0.88, 95%CI: [0.81; 0.96], *p*-value: 0.002). Patients who had one or more hospitalization in the year preceding inclusion were less likely to be non-adherent compared to those with no hospitalization at all (OR = 0.40, 95%CI: [0.17; 0.96], *p*-value: 0.04) (Table 3).

Concerning the patterns of forgoing healthcare between OSAS and CRF, the four most frequent types of healthcare foregone were consultations with specialists (51.5%), purchase of medical equipment (35.4%), consultations with primary care physicians (30.3%) and dental care (28.3%) (Supplementary Table 2).

**Figure 1:**
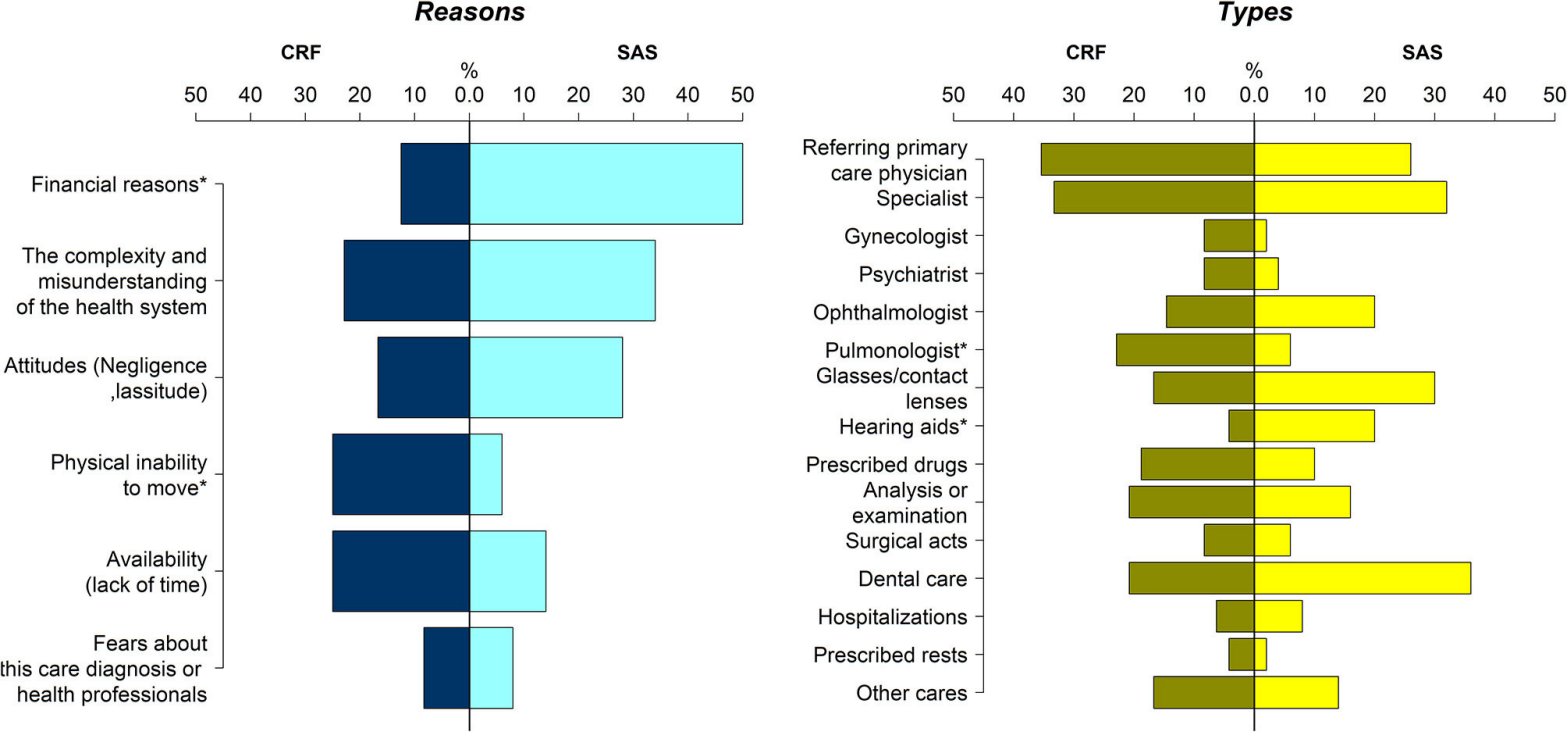
Differences in the pattern of healthcare non-take up between OSAS and CRF patients. The rate of each type of healthcare type forgone and the reasons of the non-take up are presented as percentages (%).

**Figure 2:**
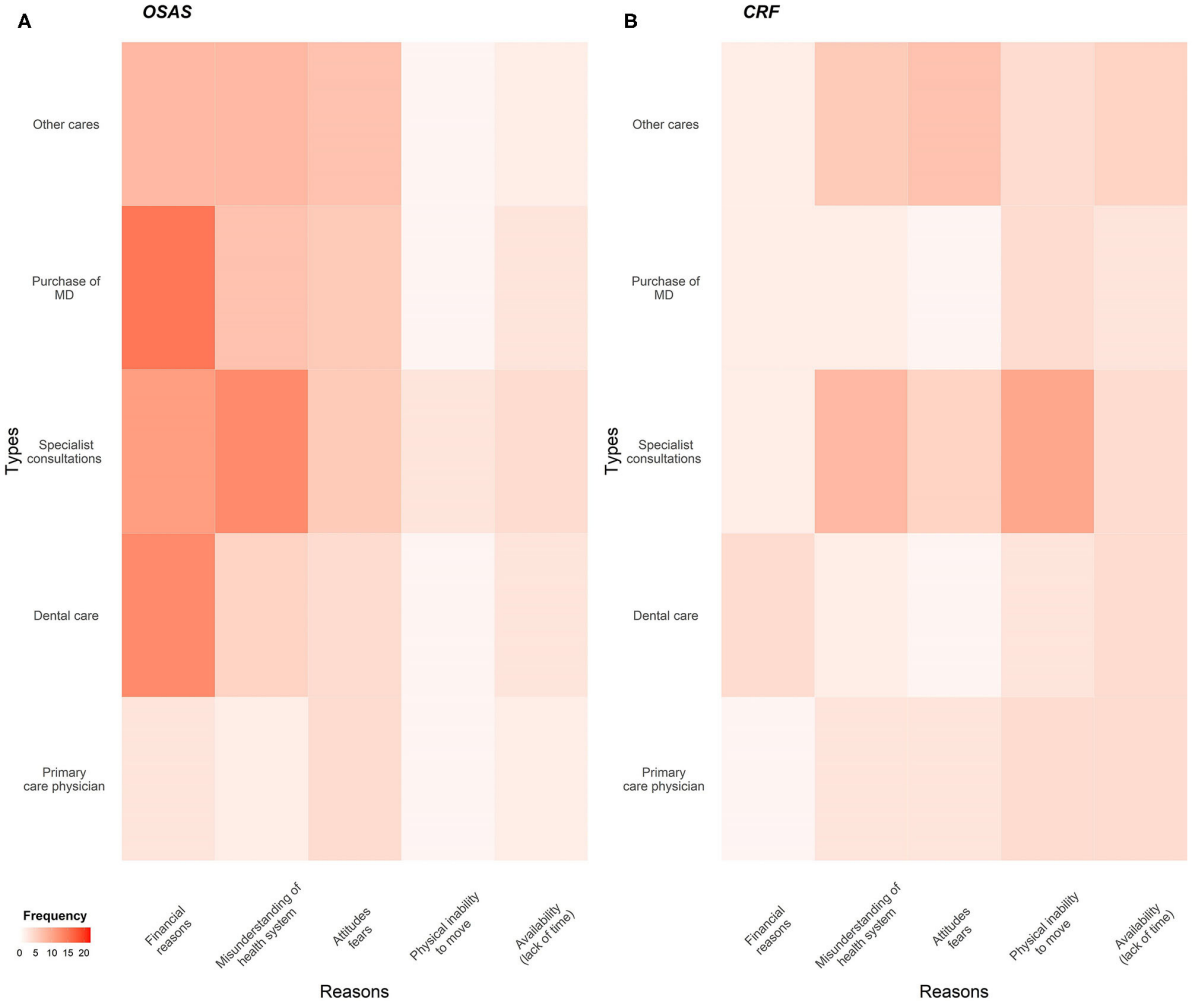
Heatmap displaying the types and reasons of healthcare non-take up. **(A)** OSAS patients; **(B)** CRF patients. MD, medical device.

Although the rate of forgoing healthcare was not different between OSAS and CRF (respectively 32.3% vs. 35.3%, *p* = 0.59; Table 3), the reasons for forgoing care were significantly different. For patients with OSAS it was mainly for financial reasons (49% vs. 12.5% in CRF group, *p* < 0.01), whereas patients with CRF forwent healthcare due to lack of mobility (25% vs. 5.9% in sleep apnea group, *p* = 0 < 0.01). Figure 1 shows the types of healthcare forgone and reasons. Figure 2 links types and reasons. In patients with CRF (2b), the lack of mobility was strongly linked to forgoing specialist consultations. In contrast, mainly financial reasons were given by the OSAS group (2a) (Figures 1, 2, and Supplementary Table 2).

## Discussion

This study investigated the relationship between patterns of non-uptake of healthcare and PAP therapy adherence in two distinctive populations, OSAS and CRF. The rate of forgoing healthcare was higher (33.7%) than that reported in the general French population (25.4%) (23). Deprivation and foregoing healthcare exert a synergistic effect, increasing the risk of being non-adherent to PAP therapies. The picture was different in OSAS and CRF patients reflecting the functioning of the French healthcare system.

As identified in previous studies (13, 31), our results show a significant association between the level of multidimensional deprivation and PAP adherence. The novelty of our findings is to demonstrate that the combination of deprivation and forgoing healthcare is associated with a nearly 8-fold higher risk of being non-adherent. This reflects the complexity and multi-dimensionality of PAP adherence issues and the need for more transdisciplinary approaches to understand how these several social factors interact (32). The known determinants of low adherence are poorly informative explaining the 4 to 25% of variance in PAP adherence (33). There is a need to include a systematic assessment of deprivation and healthcare non-take up using appropriate questionnaires at the time of PAP therapy initiation. The consideration of societal topics should be better addressed in the education of sleep and respiratory physicians. Additionally, studies are needed to investigate the impact of health policy interventions on PAP adherence, as has been done for medications in vulnerable populations (34).

The inclusion of patients with a variety of respiratory diseases requiring PAP therapies is another originality of our study. The subsets of patients with OSAS and CFR had different patterns of types and reasons for forgoing healthcare. This reflects both different socio-economic circumstances and different health insurance coverage for the respective underlying disease. OSAS patients, with public system coverage limited to 60%, declared forgoing healthcare mainly for financial reasons whereas CRF patients (100% public coverage) explained forgoing healthcare mainly due to their lack of mobility.

For individuals with CRF the total reimbursement of healthcare costs by the French state, potentially makes it possible to totally eliminate financial barriers to healthcare access. However, the physical and psychological disabilities of CRF have repercussions leading to a deterioration in quality of life and loss of autonomy (difficulty to move about and/or the need of assistance) (35). This underlines the need for tailored solutions with an extension of public coverage to a subset of OSAS cases and greater use of telemedicine to preserve the continuity of care for CRF patients (36, 37).

In OSAS patients having only partial (60%) cover, a clear renunciation of dental and ophthalmic care was found. This has recently been addressed in France by the implementation of universal full reimbursement (“Rest à charge 0” [Zero cost to patient]) of basic dental care and glasses.

While our study is unique, it also has limitations. The main one being that we included patients treated with PAP therapies for at least 1 year whereas the mean duration of PAP treatment exceeds 8 years. This restricted the subgroup of non-adherent patients and potentially the power of the study to demonstrate an even greater effect of health care non-take-up on adherence. Further studies are needed to investigate the impact of health care non-take-up on initial PAP refusal and early PAP termination. Secondly, the present results did not consider comorbidities and polypharmacy that may be associated with healthcare non-take-up and PAP adherence (38). Finally, our study allowed to compare the healthcare non-take-up profile between the CFR and OSAS population using an explanatory exploratory approach. The aim was to obtain assumption for further research and not to provide conclusions based on a study which was not designed for this purpose. In conclusion, our study provides unique data indicating how the quality of care in PAP therapies could be improved and the design of interventional studies tailored to types and reasons for forgoing healthcare.

## Data Availability Statement

The raw data supporting the conclusions of this article will be made available by the authors, without undue reservation.

## Ethics Statement

The studies involving human participants were reviewed and approved by French Ethics Committee Ile de France II. The patients/participants provided their written informed consent to participate in this study.

## Author Contributions

J-LP, MJ-J, SB and J-CB designed the study. ND collected the data. ND and SB carried out the statistical analyses and produced the figures. ND, J-CB, SB, AF, RT, and HR interpreted the data. ND, J-CB, SB and AF wrote the manuscript. RT, HR, MJ-J and J-LP revised the manuscript. All authors approved the version to be submitted for publication and took responsibility for the integrity of the work as a whole.

## Conflict of Interest

J-CB and ND are employees of AGIR a dom. Homecare charity. The remaining authors declare that the research was conducted in the absence of any commercial or financial relationships that could be construed as a potential conflict of interest.

## Publisher's Note

All claims expressed in this article are solely those of the authors and do not necessarily represent those of their affiliated organizations, or those of the publisher, the editors and the reviewers. Any product that may be evaluated in this article, or claim that may be made by its manufacturer, is not guaranteed or endorsed by the publisher.

## Abbreviations

AHI: Apnea hypopnea index
BMI: Body mass index
CPAP: Continuous Positive Airway Pressure
CRF: Chronic respiratory failure
CSS: Complémentaire Santé Solidaire (state-subsidized complementary (top-up) insurance)
EPICES: Evaluation de la précarité et des inégalités de santé dans les Centres d'examens de santé (Assessment of precariousness and health inequalities in health examination centers)
ESS: Epworth sleepiness score
IQR: Interquartile range
NIV: Non invasive ventilation
OR: Odds Ratio
OSAS: Obstructive Sleep Apnea Syndrome
PAP: Positive Airway Pressure
SD: Standard Deviation.
