# Supplementary material for: Impact of Healthcare Non-Take-Up on Adherence to Long-Term Positive Airway Pressure Therapy
Source: Front Public Health. 2021 Aug 17;9:713313. doi: 10.3389/fpubh.2021.713313 (PMC8416102; doi:10.3389/fpubh.2021.713313)
Supplement: Supplementary file 1 [file Data_Sheet_1.docx]

# **e-Figure 1: Study design**

**
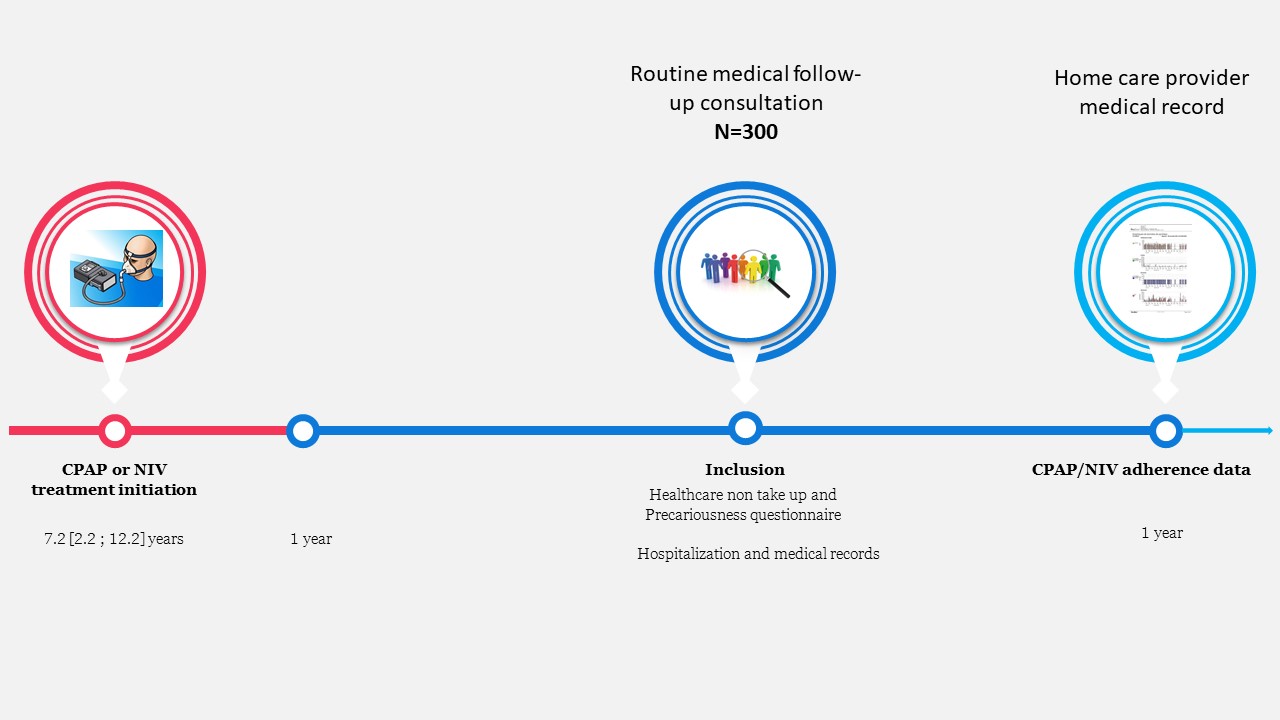
**

**e-Figure 2: Healthcare non-take up questionnaire (BRS)**

*(Developed and administered in French)*


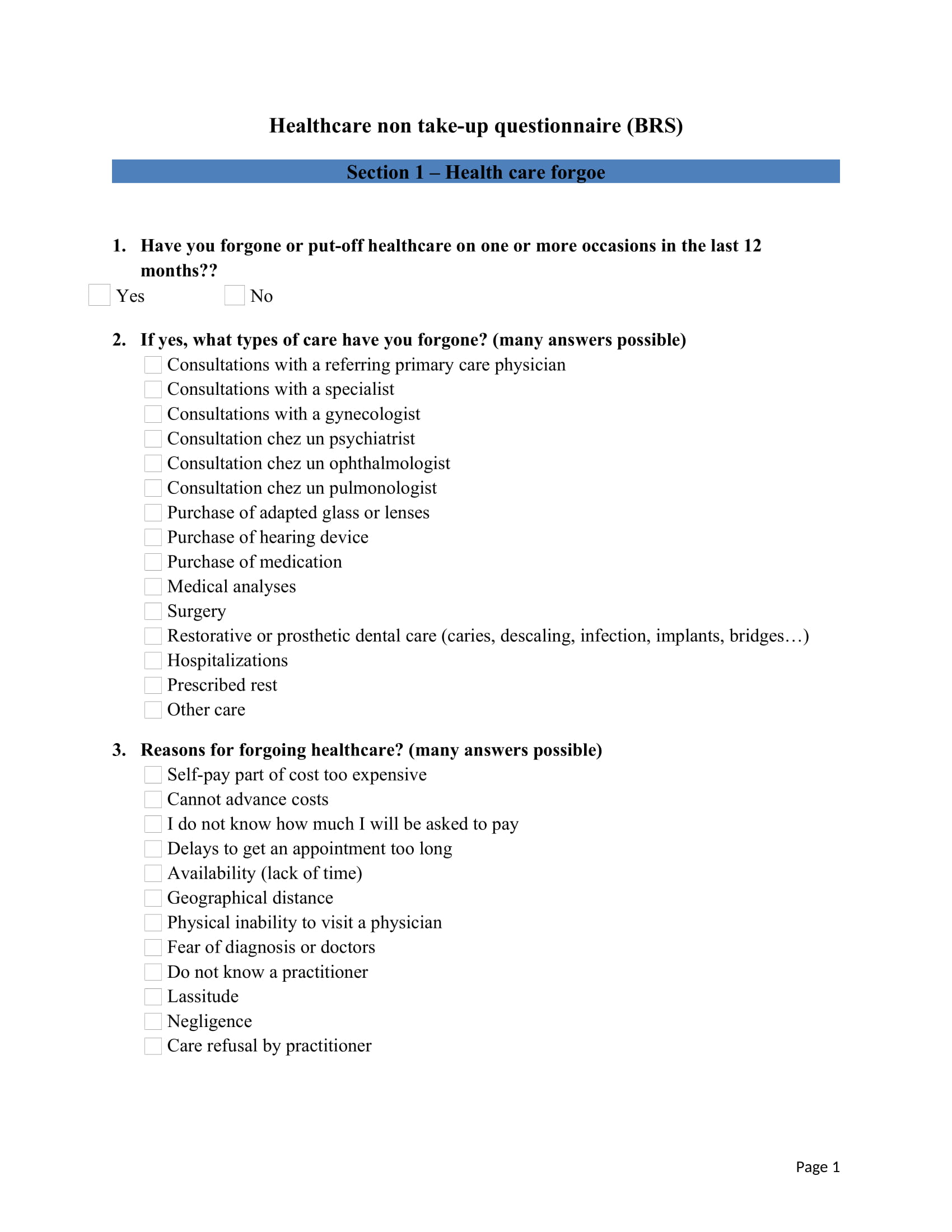

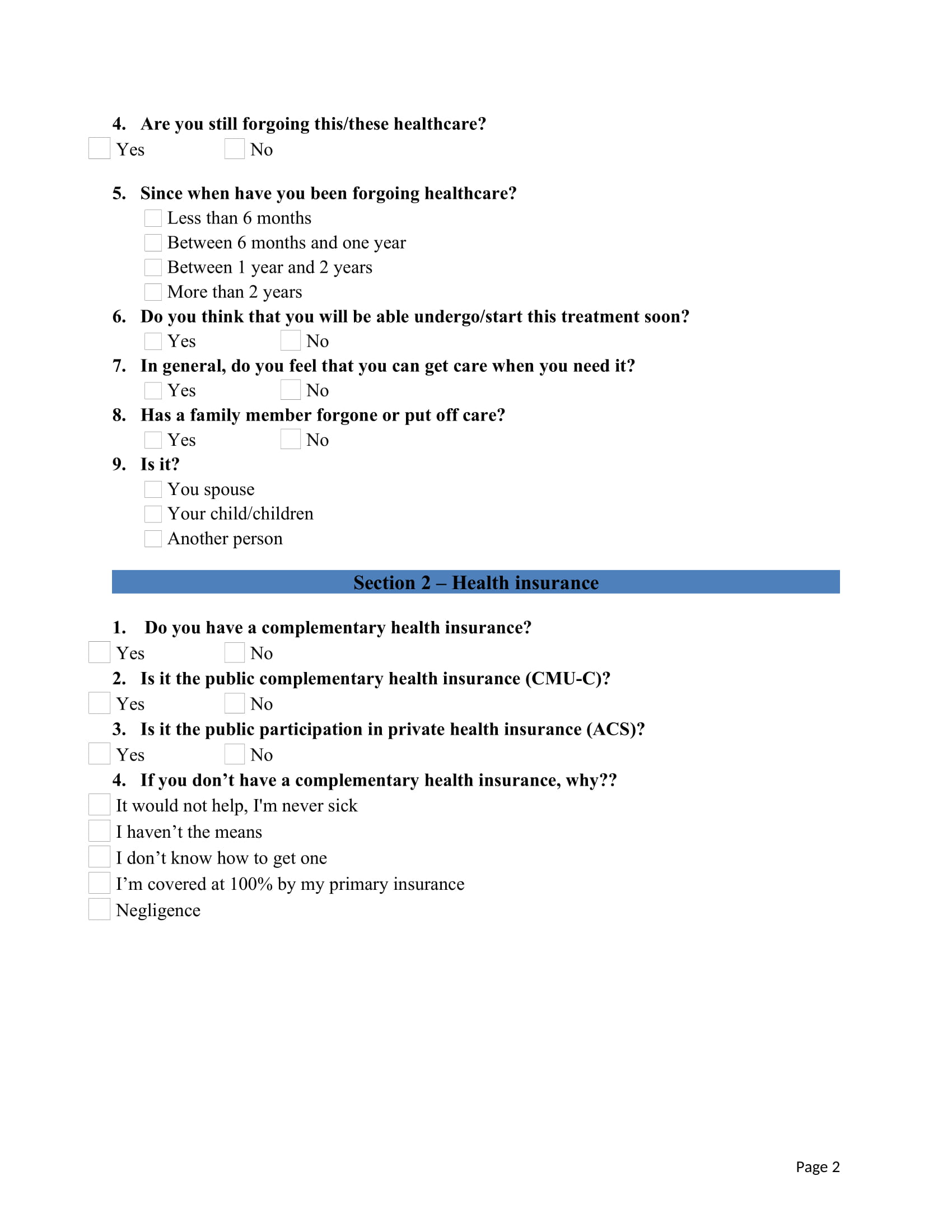

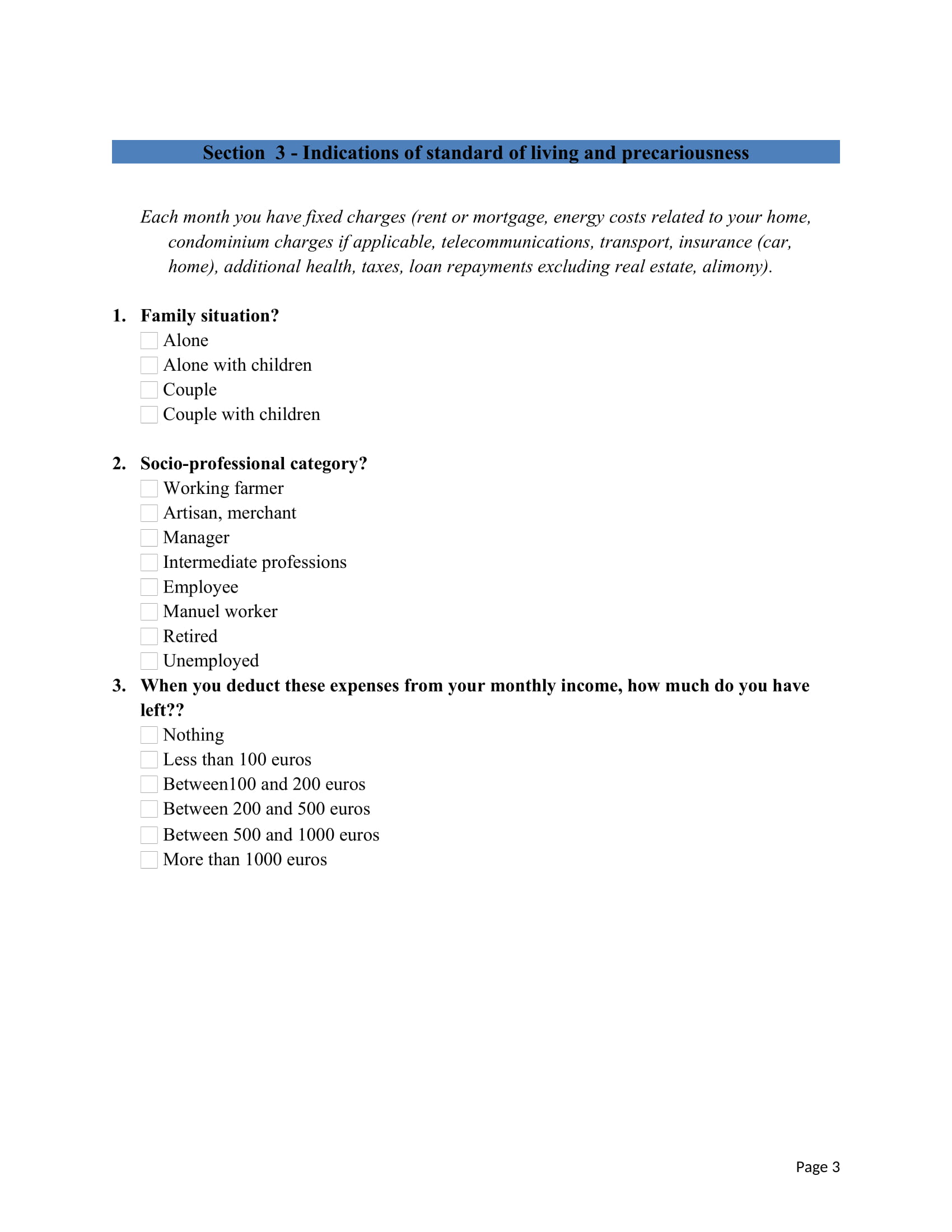

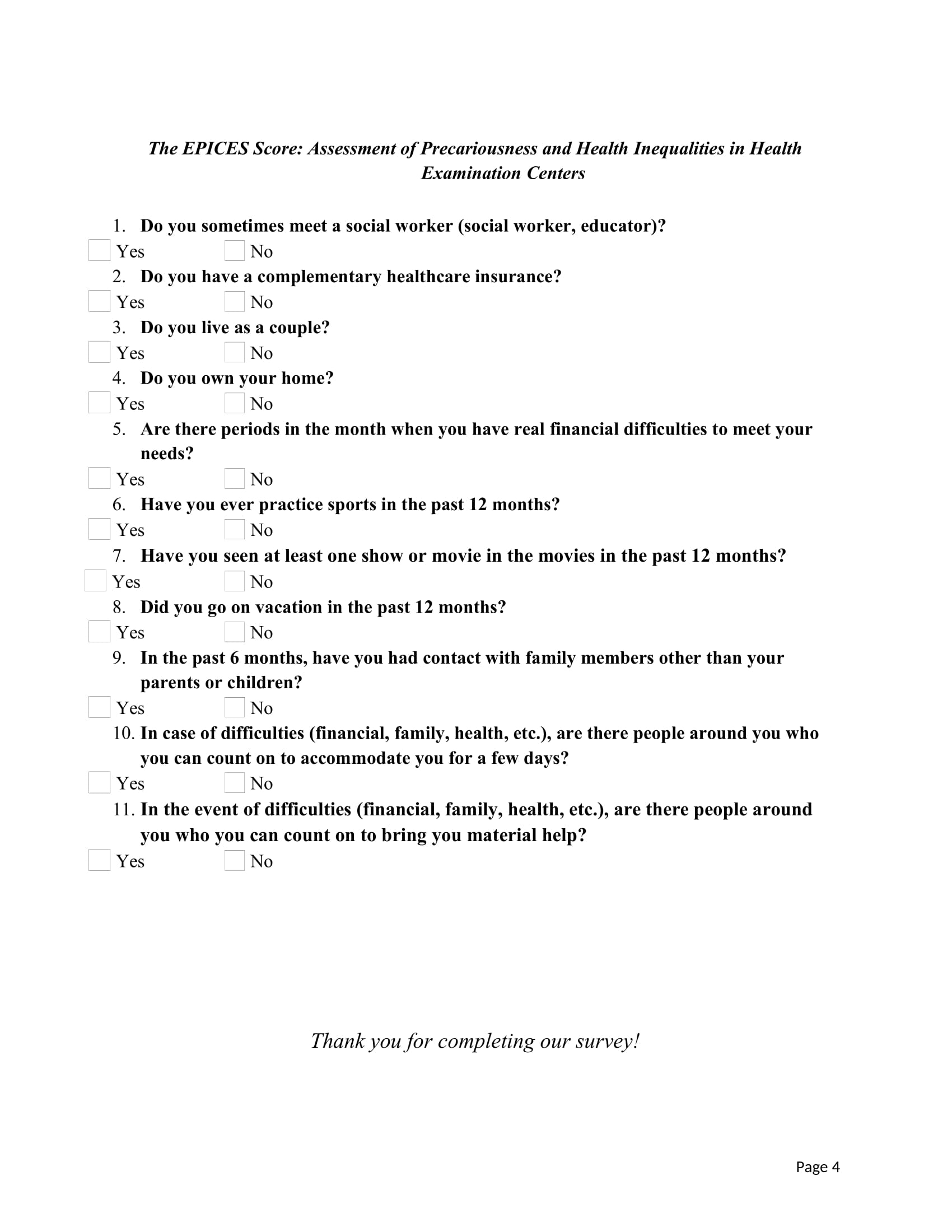


# **e-Figure 3: Flowchart of the study**

298 CPAP/NIV/O_2_ treated patients who completed the healthcare non-take up questionnaire

No healthcare forgone

N=195 (66.3%)

Healthcare forgone

N=99 (33.7%)

- 4 patients did not respond to the key healthcare non-take up question

294 CPAP/NIV/O_2_ treated patients who responded to the key healthcare non-take up question

150 NIV/O_2_ treated patients who completed the healthcare non-take up questionnaire

148 CPAP treated patients who completed the healthcare non-take up questionnaire

# **e-Table 1: Univariable analysis of factors associated with the probability of being non-adherent (logistic regression)**

| **Variable** | **Items** | **OR** | **95% CI** | **p-value** |
| --- | --- | --- | --- | --- |
| Sex | F | 1.47 | [0.70 ; 3.06] | 0.31 |
|  | M | REF |  |  |
| Age | <=60 years | 1.39 | [0.57 ; 3.34] | 0.47 |
|  | ]60 ; 70] | 0.90 | [0.37 ; 2.19] | 0.81 |
|  | >70 years | REF |  |  |
| BMI (Kg/m^2^) |  | 0.98 | [0.93 ; 1.02] | 0.27 |
| Family situation | Couple | 1.06 | [0.51 ; 2.21] | 0.87 |
|  | Alone | REF |  |  |
| Socio-professional situation | In employment | 1.48 | [0.66 ; 3.29] | 0.34 |
|  | Retired or Unemployed | REF |  |  |
| Complementary healthcare insurance | None or CSS | 1.62 | [0.65 ; 4.03] | 0.30 |
|  | Private | REF |  |  |
| LDD | Long-term Chronic disease / Total Disability | 1.69 | [0.78 ; 3.71] | 0.19 |
|  | NO | REF |  |  |
| Healthcare forgone | NO | 0.34 | [0.16 ; 0.71] | <.01 |
|  | Yes | REF |  |  |
| EPICES score | EPICES score >30 | 4.13 | [1.73 ; 9.87] | <.01 |
|  | EPICES score <=30 | REF |  |  |
| CPAP/NIV equipment duration |  | 0.87 | [0.81 ; 0.94] | <.01 |
| Number of hospitalizations in the last 12 months | None | 2.10 | [0.94 ; 4.71] | 0.07 |
|  | One or more hospitalization | REF |  |  |
| Pathology | Chronic respiratory failure | 1.08 | [0.52 ; 2.23] | 0.84 |
|  | OSAS | REF |  |  |

**e-Table 2: Patterns of healthcare non-take up (N=47):**

| **Variable** | **Items** | **Whole population (N=99)** | **SAS**  **(N=48, 48.48%)** | **CRF**  **(N=51, 52.26 %)** | **p-value** | **Missing** |
| --- | --- | --- | --- | --- | --- | --- |
| **Types of healthcare forgone** |  |  |  |  | **.** | **.** |
| ***Consultation with primary care physician*** | YES | 30 (30.3) | 13 (25.5) | 17 (35.4) | 0.28 | 0 |
| ***Dental care*** | YES | 28 (28.3) | 18 (35.3) | 10 (20.8) | 0.11 | 0 |
| ***Specialist consultations*** | YES | 51 (51.5) | 24 (47.1) | 27 (56.3) | 0.36 | 0 |
| - Other specialist | YES | 32 (32.3) | 16 (31.4) | 16 (33.3) | 0.83 | 0 |
| - Gynecologist | YES | 5 (5.1) | 1 (2) | 4 (8.3) | 0.15 | 0 |
| - Psychiatrist | YES | 6 (6.1) | 2 (3.9) | 4 (8.3) | 0.36 | . |
| - Ophthalmologist | YES | 17 (17.2) | 10 (19.6) | 7 (14.6) | 0.51 | . |
| - Pulmonologist | YES | 14 (14.1) | 3 (5.9) | 11 (22.9) | 0.02 | . |
| ***Purchase of medical equipment and drugs*** | YES | 35 (35.4) | 22 (43.1) | 13 (27.1) | 0.09 | . |
| - Glasses or contact lenses | YES | 23 (23.2) | 15 (29.4) | 8 (16.7) | 0.13 | . |
| - Hearing aids | YES | 12 (12.1) | 10 (19.6) | 2 (4.2) | 0.02 | . |
| - Prescribed drugs | YES | 14 (14.1) | 5 (9.8) | 9 (18.8) | 0.20 | 0 |
| ***Other care*** | YES | 40 (40.4) | 18 (35.3) | 22 (45.8) | 0.29 | 0 |
| - Analysis or examination | YES | 19 (19.2) | 9 (17.6) | 10 (20.8) | 0.69 | 0 |
| - Surgical acts | YES | 7 (7.1) | 3 (5.9) | 4 (8.3) | 0.63 | 0 |
| - Hospitalizations | YES | 7 (7.1) | 4 (7.8) | 3 (6.3) | 0.76 | 0 |
| - Prescribed rest | YES | 3 (3) | 1 (2) | 2 (4.2) | 0.52 | 0 |
| - Other | YES | 15 (15.2) | 7 (13.7) | 8 (16.7) | 0.68 | 0 |
| **Reasons for forgoing healthcare** |  |  |  |  |  |  |
| ***Financial reasons :*** | YES | 31 (31.3) | 25 (49) | 6 (12.5) | <.01 | 0 |
| - Self-pay part of costs too expensive | YES | 23 (23.2) | 18 (35.3) | 5 (10.4) | <.01 | 0 |
| - Advance of costs not possible | YES | 17 (17.2) | 14 (27.5) | 3 (6.3) | <.01 | 0 |
| - I do not know how much I will be asked to pay for this care | YES | 14 (14.1) | 9 (17.6) | 5 (10.4) | 0.30 | 0 |
| ***The complexity and misunderstanding of the system of health insurance:*** | YES | 28 (28.3) | 17 (33.3) | 11 (22.9) | 0.25 | 0 |
| - Delays for appointment too long | YES | 20 (20.2) | 13 (25.5) | 7 (14.6) | 0.18 | 0 |
| - Geographical distance | YES | 7 (7.1) | 3 (5.9) | 4 (8.3) | 0.63 | 0 |
| - No knowledge of a where to find a practitioner | YES | 2 (2) | 1 (2) | 1 (2.1) | 0.97 | 0 |
| - Care refusal by practitioner (saturation, discrimination) | YES | 4 (4) | 3 (5.9) | 1 (2.1) | 0.34 | 0 |
| ***Attitudes*** | YES | 23 (23.2) | 15 (29.4) | 8 (16.7) | 0.13 | 0 |
| - Lassitude | YES | 9 (9.1) | 4 (7.8) | 5 (10.4) | 0.66 | 0 |
| - Negligence | YES | 15 (15.2) | 11 (21.6) | 4 (8.3) | 0.07 | 0 |
| - Fear about this diagnosis / of health professionals | YES | 8 (8.1) | 4 (7.8) | 4 (8.3) | 0.93 | 0 |
| ***Lack of mobility*** | YES | 15 (15.2) | 3 (5.9) | 12 (25) | <.01 | 0 |
| ***Availability (lack of time)*** | YES | 19 (19.2) | 7 (13.7) | 12 (25) | 0.15 | 0 |
| Are you still forgoing this care? | YES | 41 (43.6) | 22 (44.9) | 19 (42.2) | 0.79 | 5 |
| Since when have you been forgoing healthcare | Between 1 year and 2 years | 11 (15.3) | 8 (20) | 3 (9.4) | 0.15 | 27 |
|  | Between 6 months and 1 year | 15 (20.8) | 11 (27.5) | 4 (12.5) | . | . |
|  | Less than 6 months | 20 (27.8) | 10 (25) | 10 (31.3) | . | . |
|  | More than 2 years | 26 (36.1) | 11 (27.5) | 15 (46.9) | . | . |
| Do you think that you will be able undergo/start this treatment soon? | YES | 51 (68) | 28 (70) | 23 (65.7) | 0.69 | 24 |
| Does another member of your family forgo healthcare? | YES | 25 (29.8) | 13 (29.5) | 12 (30) | 0.96 | 15 |
| If so, who? | Other | 7 (26.9) | 3 (23.1) | 4 (30.8) | 0.23 | 73 |
|  | Your partner | 12 (46.2) | 7 (53.8) | 5 (38.5) | . | . |
|  | Your partner & children | 2 (7.7) | 2 (15.4) | 0 (0) | . | . |
|  | Your children | 5 (19.2) | 1 (7.7) | 4 (30.8) | . | . |

*Values in Numbers (%) or median*
